# Supplementary material for: Codesign and Feasibility Testing of a Tool to Evaluate Overweight and Obesity Apps
Source: Int J Environ Res Public Health. 2022 Apr 28;19(9):5387. doi: 10.3390/ijerph19095387 (PMC9103883; doi:10.3390/ijerph19095387)
Supplement: Supplementary file 1 [file ijerph-19-05387-s001.zip › ijerph-1627979-supplementary.pdf]

ANNEX 1: PROTOCOL OF THE COCREATION WORKSHOP OF THE EVALAPPS PROJECT

DESCRIPTION OF THE WORKSHOP

Date: November 22th 2018
Duration: 3 hours
Attendees: between 6 and 10 people
Profile:

- Healthcare professionals
- Experts in technological development
- Teaching experts (UOC teachers)
- Researchers

Objective: To define the characteristics and contents of the EVALAPPS tool.

BACKGROUND

The sesión is embeded in the "Design Thinking" process.
Prior to the workshop, both, as systematic review and a Delphi analysis were undertaken, responding to the "empathize" and "define" phases.
The sesión aims to collectively devise the tool.
After the workshop, a developer will be in charge of designing an application prototype that will be tested through a pilot study with end-users.

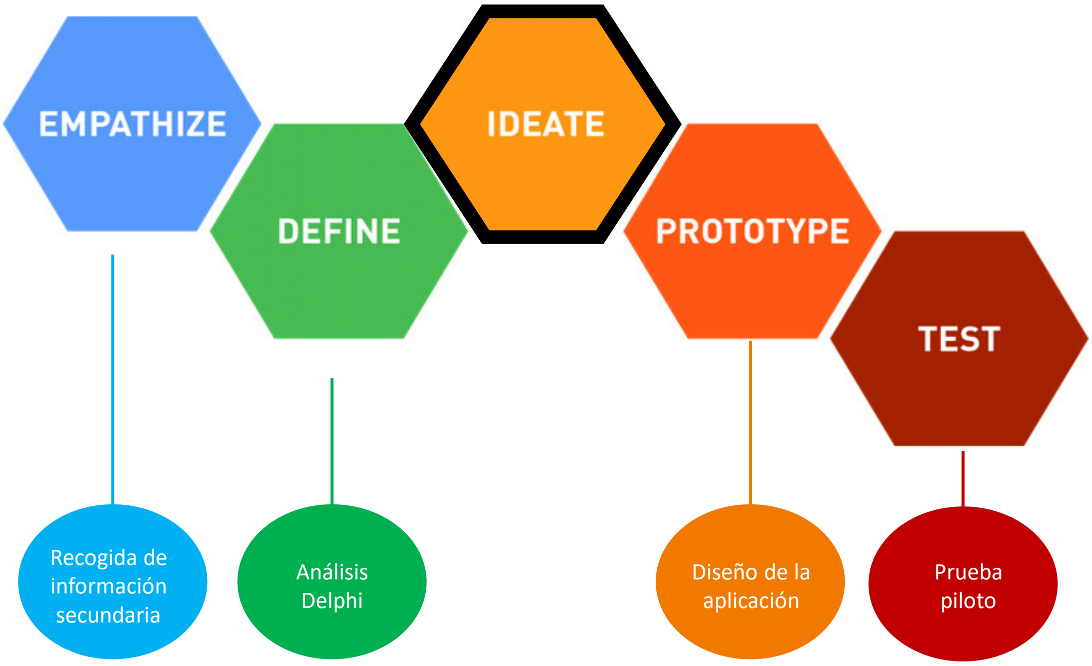

DEVELOPMENT OF THE WORKSHOP

Welcome and presentation

1. Brief explanation of the project by the principal investigator (objectives, members of the project, actions carried out...)
2. Contextualization of the sesión by the facilitator (objectives, expected time, structure of the workshop and topics to be addressed) making special emphasis on:

- Throughout the workshop we will pose a series of challenges to be solved, and we will combine the work in both small and large groups for their resolution.
- The objective of the workshop is to collect your opinions, without judging the content.
- There are no good or bad answers, all opinions are useful and can help improve the tool we want to develop .

Questions and doubt solvin¡g time (5-10 minutes).
4. Distribution of the consent form and collection of participants signatures.
5. A round of presentation of the participants (name, profession, occupation and relationship with the theme) is opened.

First challenge: profiles

The facilitator proposes to the group the first question to be discussed: The first challenge is to decide whether there should be a single profile or different profiles of evaluators

A flipchart paper is placed on the table with the two possible options (one profile vs. Different profiles) and a sticker is given to each participant.

In silence, each participant must place their sticker on the option they consider most appropriate. In case someone considers that the option 'two or more profiles' he or she is asked to specify the profiles (health professionals, users, application developers...)

Green and red post-its are distributed to participants toidentify the advantages (green post-it) and disadvantages (red post-it) of each alternative. Afterwards, a debate is opened to collect the different opinions.

Un único perfil		Dos o más perfiles	
Ventajas	Desventajas	Ventajas	Desventajas

Second challenge. Introduction to the evaluation

The facilitator proposes to the group the second challenge: How the result of the evaluation of the applications should be introduced into the tool

Participants are divided into small groups of 2-3 people. If in the previous debate it has been concluded that there should be more than one profile, the functions are distributed among the groups:

- Health professionals will be responsible for developing the evaluation for the professional profile
- The people who could represent users of the application, will be responsible for developing the evaluation for the user profile
- If a developer profile has been identified, for example, the developers will be entrusted with this task
- The rest of the participants will be distributed in a balanced way among the different groups

If a single profile has been defined all groups develop the application in parallel

The following support material to "design" the application is distributed to the participants:

Sheets DIN-A3 sheets

Markers

List of dimensions and criteria agreed in the Delphi previous analysis. The list with all the dimensions is distributed to all the groups indicating that it is a basic documentation that must be used to carry out the exercise, but indicating that not all the dimensions have to be covered in detail but only those that they consider relevant.

Script of questions to guide reflection

The facilitator indicates that each DIN-A3 sheet is equivalent to an application screen, and the following instructions are given:

Each DIN-A3 sheet is a screen

For time reasons, we are not going to ask you to go into the detail of the criteria to be applied. What interests us is that you work on the general design of the tool (what dimensions of the list that we have proposed should be evaluated, in what order, all are mandatory, how the questions are posed, ...).

If you have time, we are also interested in you thinking about the "look and feel" of the application, but it is not a priority

As you develop your "screens", hang them on the wall sequentially, simulating the design of the application.

When all participants have defined the application, the work done is pooled

Third challenge. Evaluation result

The facilitator proposes to the group the third challenge whic is the agreement on how the results introduced during the evaluation should be interpreted. Should all dimensions have equal weight?

If there is no agreement on this question and the participants consider that the dimensions should have different weights, a sheet with the list of dimensions is distributed to the participants and they are explained that they have € 100 that they must distribute, as they want, among the dimensions (all in one dimension, in equal parts, in different quantities, only in some dimensions...).

	Weight		
	High	Medium	Low
Dimension 1			
Dimension 2			
Dimension 3			
…			

The result of the vote is commented on and the qualitative assessments of the participants are collected.

fourth challenge: Presentation of the resultS

The facilitator poses the fourth challenge of the workshop: Definition on how the results should be displayed to the user.

A debate opens up about whether it should be differentiated according to the type of user (patient, healthcare professional, manager?).

The different alternatives are presented and a round of debate is opened to assess the possible options

Workshop ending

The principal investigator thanks the work done and informs of the next steps

SUPPORT MATERIALS TO BE USED DURING THE WORKSHOP

LIST OF DIMENSIONS
App purpose: Monitoring, guidance and/or treatment
Security and privacy: Security and privacy standards of user's data
Clinical effectiveness: Degree of scientific evidence on which the app is based
Information and content quality: Reliability and credibility of the contents of the app
Usability: Adherence, entertainment, personalization, interactivity and user experience
Funcionalities: Ease of use and navigation
Level of development: Level of interoperability achieved
Health indicators - Personal data: Data collected about the user
Health indicators - Physical condition data: Data collected on the physical condition of the user
Health indicators - Activity data: Data collected about the activity carried out by the user

Posible questions to be asked during the sesion to generate debate

How do we access the evaluation tool?
Can I enter without a log in or do I need a log in? What information is asked of the user to make this log in?

How to access the app you want to evaluate?
Should the name of the app be typed? Will there be a repository/list to choose which app to evaluate? Will only those pending assessment be available?

What dimensions should be evaluated?
Should all dimensions be evaluated? Or specific dimensions (which)? Should everyone assess the same dimensions? Or should different dimensions be evaluated based on the person's profile?

How should the evaluation be structured?
Should it be a list of criteria, without specifying what dimension they belong to? Or should they be classified by dimensions, so that the person doing the evaluation is aware of what dimension they is being evaluated?

In what order should the evaluation questions be asked?
Is it important that there is an order? If so, what should it be? Based on what criteria should be established?

What information should be given to the user?
Do I have to indicate the volume of criteria to be evaluated (either in number, or approximate time it will take to carry out the evaluation)?. Is it necessary to define the dimensions and/or evaluation criteria? so that the person knows what is being evaluated specifically? When should this information appear? Is it necessary to give an explanation of how each criterion is evaluated?

What format should the valuation have?
Likert Ladder, Yes/No, open questions, color scale, stars ...? Should it always be the same format or combine different types of questions? Is it allowed to enter comments? In this case, at what point: on each screen, at the end of everything?

How do we browse the app?
Do we move with a scroll, with all the criteria and dimensions listed on the same screen? at the discretion per screen, for groups of 2-3 criteria per screen, by dimensions? What "buttons" should be incorporated into the application (forward, back, home, print...)? Do I need to enter any element that indicates when the evaluation remains to be completed?

Annex 2. Missing and valid information of the EVALAPPS Pilot testing.

		Missing values		Valid information	
**Criteria grouped by dimensions**		N	%	N	%
**App purpose**					
Is it clearly explained what the App is for?		0	0.0	24	100
Does the content of the App correspond to the description that appears in the application store (Google Play or iTunes)?		2	8.33	22	91.67
Do you think that the app responds to the objective for which it was created?		2	8.33	22	91.67
**Development**					
Is the App available in iOS and Android?		2	8.33	22	91.67
Does the App allow printing / downloading the collected data?		3	12.50	21	87.50
Does the App allow you to send data to your medical doctor so that it can be added to your clinical record?		4	16.67	20	83.33
**Reliability**					
Does the App provide information on who has developed it and the participation of third parties?		4	16.67	20	83.33
Do App developers generate trust / credibility?		4	16.67	20	83.33
In case the App sells products / services: does it provide clear information about the commercial conditions?		4	16.67	20	83.33
Does the App send any alert in case it detects risky behaviors? (for example, very strict diets)		4	16.67	20	83.33
Are the contents offered by the App supported by a scientific study or a report that validates its quality?		4	16.67	20	83.33
**Usability and functionality**					
Does the App have a friendly and intuitive interface?		4	16.67	20	83.33
Are the information and the instructions of the App easily understood?		5	20.83	19	79.17
Is it clear what needs to be done in each moment?		6	25.00	18	75.00
Is the registration form of your data (weight, age, physical activity, ...) easy to use?		7	29.17	17	70.83
Is navigation through the different screens of the App easy, fast and intuitive?		7	29.17	17	70.83
Do the components of the App (menus / buttons) work quickly?		7	29.17	17	70.83
Are the icons and buttons of the App easily interpretable and understandable, and clearly reflect the associated features?		7	29.17	17	70.83
**Health indicators**					
Does the App allow you to record your weight?		8	33.33	16	66.67
Does the App allow you to record your age?		8	33.33	16	66.67
Does the App allow you to record your height?		9	37.50	15	62.50
Does the App allow you to record the type of physical activity you do?		9	37.50	15	62.50
Does the App allow you to record the times per week that you do physical activity?		10	41.67	14	58.33
Does the App record the time you spend doing physical activity per day / week?		11	45.83	13	54.17
Can the App collect information about sedentary habits day / week? (for example, hours of sitting time, hours of screentime)		11	45.83	13	54.17
Can the App record the number of times you eat per day?		12	50.00	12	50.00
Can the App record the type of food you eat per day?		12	50.00	12	50.00
Can the App record the amount of food you eat per day?		12	50.00	12	50.00
If the App allows you to record body measurements (weight, thoracic circumference, abdominal perimeter, ...): can these be entered repeatedly to monitor their evolution?		12	50.00	12	50.00
Does the App allow you to collect data about your sleeping hours?		13	54.17	11	45.83
Does the App allow you to collect data about your medical history? (previous diseases, medical tests, etc.)		13	54.17	11	45.83
**Clinical effectiveness**					
Are the health recommendations suggested by the App based on scientific evidence (clinical studies, recommendation guides, etc.)?1		13	54.17	11	45.83
Is the App updated periodically according to the new scientific evidence?		13	54.17	11	45.83
Is it specified in what information sources is the App based? (clinical studies, recommendation guides, etc.		13	54.17	11	45.83
Does the App have the endorsement of any scientific society, health organization or Ministry of Health?		13	54.17	11	45.83
Does the App propose measurable and achievable objectives?		13	54.17	11	45.83
**Security and privacy**					

Does the App inform in a clear and simple way about the legal regulations related to the protection of personal data?		13	54.17	11	45.83
Is it clear who is the owner of the data?		13	54.17	11	45.83
Can the user ask the provider to delete the App and his/her data?		13	54.17	11	45.83
Does the App inform about the terms and conditions of use and guarantees the security?		13	54.17	11	45.83
Does the App clearly inform about data cessation with tthird parties?		13	54.17	11	45.83
Can the user choose not to share data with third parties?		13	54.17	11	45.83
Does the App inform if data is encrypted?		12	50.00	12	50.00

Valid information: All responses eliminating ‘I have not find the information’ and ‘I don’t know’
